# Contribution of copy number variants (CNVs) to congenital, unexplained intellectual and developmental disabilities in Lebanese patients

**DOI:** 10.1186/s13039-015-0130-y

**Published:** 2015-04-09

**Authors:** Nancy Choucair, Joelle Abou Ghoch, Sandra Corbani, Pierre Cacciagli, Cecile Mignon-Ravix, Nabiha Salem, Nadine Jalkh, Sandra El Sabbagh, Ali Fawaz, Tony Ibrahim, Laurent Villard, André Mégarbané, Eliane Chouery

**Affiliations:** Unité de Génétique Médicale et Laboratoire Associé INSERM à l’Unité UMR_S 910, Faculté de Médecine, Université Saint-Joseph, Beirut, Lebanon; Faculté de Médecine de la Timone, Aix-Marseille Université, Marseille, France; Institut National de la Santé et de la Recherche Médicale, UMR_S910, Marseille, France; Département de Génétique Médicale, Assitance Publique Hôpitaux de Marseille, Hôpital d’Enfants de La Timone, Marseille, France; Service de Pédiatrie, Hotel Dieu de France Hospital, Beirut, Lebanon; Neuropediatrics Department, Lebanese University, Beirut, Lebanon; Département de Médecine interne, Hotel Dieu de France Hospital, Beirut, Lebanon; Institut Jérôme Lejeune, Paris, France; Université Saint-Joseph, rue de Damas B.P. 17-5208 Mar Mikhael, Beyrouth, 11042020 Lebanon

**Keywords:** Affymetrix 2.7 M, Affymetrix 6.0, Consanguinity, Copy number variants, Database, Intellectual disability, Lebanese population

## Abstract

**Background:**

Chromosomal microarray analysis (CMA) is currently the most widely adopted clinical test for patients with unexplained intellectual disability (ID), developmental delay (DD), and congenital anomalies. Its use has revealed the capacity to detect copy number variants (CNVs), as well as regions of homozygosity, that, based on their distribution on chromosomes, indicate uniparental disomy or parental consanguinity that is suggestive of an increased probability of recessive disease.

**Results:**

We screened 149 Lebanese probands with ID/DD and 99 healthy controls using the Affymetrix Cyto 2.7 M and SNP6.0 arrays. We report all identified CNVs, which we divided into groups.

Pathogenic CNVs were identified in 12.1% of the patients. We review the genotype/phenotype correlation in a patient with a 1q44 microdeletion and refine the minimal critical regions responsible for the 10q26 and 16q monosomy syndromes.

Several likely causative CNVs were also detected, including new homozygous microdeletions (9p23p24.1, 10q25.2, and 8p23.1) in 3 patients born to consanguineous parents, involving potential candidate genes.

However, the clinical interpretation of several other CNVs remains uncertain, including a microdeletion affecting *ATRNL1*. This CNV of unknown significance was inherited from the patient’s unaffected-mother; therefore, additional ethnically matched controls must be screened to obtain enough evidence for classification of this CNV.

**Conclusion:**

This study has provided supporting evidence that whole-genome analysis is a powerful method for uncovering chromosomal imbalances, regardless of consanguinity in the parents of patients and despite the challenge presented by analyzing some CNVs.

**Electronic supplementary material:**

The online version of this article (doi:10.1186/s13039-015-0130-y) contains supplementary material, which is available to authorized users.

## Background

Intellectual disability (ID) is defined as a significant limitation in both intellectual function and adaptive behavior that originates before the age of 18 [1]. Its prevalence in the general population is estimated to be between 1 and 3%, with higher disability rates in developing countries [2-5]. In Lebanon, the latest statistical study on ID showed a relatively high incidence (4.1%) [6].

The etiological factors of ID are heterogeneous. ID with a genetic origin is more frequently found in patients with an IQ < 50 (50%) than in other patients (15%), with chromosomal aberrations being the most common causes [7,8]. Screening for these imbalances is routinely performed with conventional cytogenetic and molecular tests. Nevertheless, the resolution is limited to 5 Mb in standard karyotyping; thus, the detection of imbalances is successful only in less than 4% of patients with ID (when trisomy 21 is excluded). Other techniques such as fluorescence in situ hybridization (FISH) and multiplex ligation dependent probe amplification (MLPA), which allow the detection of microimbalances smaller than 5 Mb in targeted regions, can each explain 3% of the patients’ phenotypes [9,10].

With the introduction of the chromosomal microarray analysis (CMA) technique, which is capable of detecting submicroscopic rearrangements referred to as copy number variants (CNVs), as well as regions of homozygosity (ROH), in search for uniparental disomy or consanguinity, 10-20% of patients with unexplained ID can now be provided with a molecular diagnosis [10,11].

Herein, we report the results of a large CMA analysis project on a cohort of 149 patients with unexplained ID and a cohort of 99 controls. Our findings underscore the implication of clinically relevant CNVs in ID/DD and emphasize the ability of the technique in detecting ROH that are highly suggestive of an increased likelihood of rare recessive diseases.

## Results

In this cohort of 149 Lebanese patients having ID, DD, with or without CA, we found an average of 6 chromosomal imbalances per proband of which 67.8% had previously been reported as benign. Of the remaining CNVs, either previously reported or newly found, 10.9% were microdeletions and 21.3% were microduplications.

Twenty causal alterations (group I, Table 1) were identified in 18 patients. Eight of these pathogenic CNVs were terminal, whereas the other 12 were interstitial. One aberration, a microdeletion of 2,262 kb in patient P15, was originally determined to be a balanced translocation by standard karyotyping. Eight (8/149 or 5.3%) were greater than 5 Mb and, therefore, could have been detected by conventional karyotyping. All CNVs belonging to group I were of *de novo* origin, except for one that was inherited from the father of patient P10 [12].

**Table 1:** **Goup I, abnormal CNVs overlapping known microdeletion or microduplication syndromes and/or known pathogenic genes**

**Patient**	**Sex**	**Locus**	**Type of CNV**	**Clinical features**	**Region’s minimal size (Kb)**	**Minimal breakpoints (bp)**	**Estimated coefficient of inbreeding (F)**	**Location of the imbalance**	**Inheritance**
P1	M	1p36.33p36.22	Loss	ID, DD, Cardiac malformations, bilateral inguinal hernias, omphalocele, hearing loss, hypotrophic, triangular face, cleft lip and palate, agenesis of the corpus callosum	6,750	130,110- 136,860,002	0	Terminal	*De novo*
P2	F	1q44	Loss	ID, DD, clonus before the age of 8 months, microcephaly, growth retardation, short neck, low set ears, round face, a prominent and broad forehead, frontal bossing, small bulbous nose, anteverted nostril, deep set root, accentuated central depression lower lip, pointed chin, convergent strabismus, and midface hypoplasia , prominent supraorbital ridges, deep set eyes, dark infraorbital circles	4,293	242,895,230-247,189,052	0	Terminal	*De novo*
		6p22.3	Loss		20	15,569,409-15,589,866		Interstitial	*De novo*
P3	M	2q22.3q23.1	Loss	ID, DD, language impairment, scaphocephaly, microcephaly, autistic features, melanotic spot on belly and thighs, enophthalmia, undescended left testis, aggression	3,836	145,103,064- 148,939,789	0	Interstitial	*De novo*
P4	M	4p16.2p15.33	Loss	DD, strabismus, amblyopia, right thumb brachydactyly, missing lateral incisors	4,700	5,406,881- 10,107,795	0	Interstitial	*De novo*
P5	F	6q16.1q16.3	Loss	DD, ID, obesity, macrocephaly, strabismus, kyphosis, hyperactive, tapered fingers, genu valgum, short feet	9,622	95,014,210- 104,636,586	0	Interstitial	*De novo*
P6	M	8q24.23q24.3	Gain	DD, hypotonia, hernia diafragmatica	9,726	136,543,915- 146,270,808	1/32	Terminal	*De novo*
P7	F	9p24.3p22.3	Loss	ID, trigonocephaly, agenesis of the corpus callosum, polymicrogyria, blepharophimosis, facial dysmorphism	14,694	42,900- 14,737,134	0	Interstitial	*De novo*
P8	M	10q26.11q26.13	Loss	DD, microcephaly, language impairment, undescended right testis, micropenis, facial dysmorphism, exophthalmos, broad nasal bridge, large ears, short and flat forehead, straight eyebrows	4,570	119,502,107- 124,072,142	0	Interstitial	*De novo*
P9	F	12p12.1	Loss	ID, scaphocephaly, strabismus, camptodactyly, polymicrogyria, frontal bossing	4,260	23,572,642- 23,576,902	0	Interstitial	*De novo*
P10 [12]	F	12q24.23q24.31	Loss	DD, retrognathism, constipation, obesity, epilepsy, flat face, Café au lait spots	980	119,633,574- 120,613,673	0	Interstitial	Paternal inheritance
P11	F	14q24.3q32.2	Loss	Metatarsus adductus, enophthalmia, microretrognatism, hypotonia	23,028	75,432,536- 98,460,571	0	Interstitial	*De novo*
P12	F	15q11.2q13.1	Loss	DD, axial hypotonia, ataxia, abnormal white matter signal	5,038	21,170,573-26,208,862	0	Interstitial	*De novo*
P13	F	16p13.3	Gain	ID, short stature, renal artery stenosis, malformation of thumbs, club foot	1,558	3,057,380-4,616,365	0	Terminal	*De novo*
P14	M	16q11.2q21	Gain	Ptosis, cardiac malformation, psychomotor retardation, ID	17,756	45,027,595-62,783,676	0	Interstitial	*De novo*
P15	M	t(1;16)(q25.3;q22.3)	Loss 16q22.3	ID,DD, cerebral lesion sequelae, cleft palate	2,262	70,288,663-72,551,141	0	Interstitial	*De novo*
P16	M	22q13.2q13.33	Loss	DD, ID, hyperelastic skin of the abdomen, ligamentous laxity, pachyonychia	7,896	41,678,984-49,575,139	0	Terminal	*De novo*
P17	M	t(15;19)(q26.3;p13.3)	Loss 15q26.3	ID	992	99,225,025-100,217,472	0	Terminal	*De novo*
			Gain 19p13.3		3,124	196,466-3,321,442		Terminal	
P18	M	Xq28	Gain	ID	249	153,211,216-153,461,068	0	Terminal	*De novo*

Six CNVs were classified in group IIa (Table 2A). In 5 of these, the *de novo* or inherited status could be confirmed. Three of those CNVs were found in patients with consanguineous parents. They were homozygous and were segregated in the families. Other likely causative CNVs are *de novo* and have morbid gene like *GRHL2* and *RICTOR*.

**Table 2:** **Group IIa, rare variants likely pathogenic. Group IIb, variants of unclear significance. ND: not determined**

	**Patient**	**Sex**	**Locus**	**Type of CNV**	**Copy number state**	**Clinical features**	**Region’s minimal size (Kbp)**	**Minimal breakpoints (bp)**	**Estimated coefficient of inbreeding (F)**	**Location of the imbalance**	**Inheritance**	**Genes**
**A. Group IIa, rare variants likely pathogenic**												
Unknown CNVs but putatively pathogenic	P19	M	5p13.1	Loss	1	Ambiguous genitalia, microcephaly, seizures, bone malformations, and early death	19	39,113,442-39,132,945	0	Interstitial	ND	3 kb of *RICTOR*
	P20	M	9p24.1p23	Loss	0	Trigonocephaly, gross motor and language milestones delay, ID, a dolichocephalic pattern skull, a mild pachygyria of occipitoparietal lobes, and a mild widening of the frontal pericerebral subarachnoid space	720	8,517,597-9,238,069	1/16	Interstitial	Inherited in an autosomal recessive manner	*PTPRD*
							175	9,306,105-9,481,477				
	P21	F	8q22.3	Loss	0	Ataxia, hearing loss, ID	4	102,690,846- 102,695,425	0	Interstitial	*De novo*	*GRHL2 (intronic)*
			21q22.11	Gain	3		67	32,547,099- 32,614,769		Interstitial	*De novo*	*C21orf45*
												*MRAP*
												*URB1*
	P22	M	8p23.1	Loss	0	Syndactyly, cardiac malformation, DD, ID, ptosis,	284	8,678,807- 8,963,498	1/4	Interstitial	Inherited in an autosomal recessive manner	*ERI1, MFHAS1*
	P23	M	10q25.2	Loss	0	Psychomotor retardation, autistic features, ID	157	114,038,460- 114,196,241	1/16	Interstitial	Inherited in an autosomal recessive manner	*ACSL5*
												*ZDHHC6*
												*TECTB*
**B. Group IIb, Variants of unclear significance**												
Morbid genes inherited from a healthy parent	P24	M	10q25.3	Loss	1	Autistic behaviour, syringomielia	64	116,960,967-117,025,746	1/16	Interstitial	Maternal	*ATRNL1*

One CNV, belonging to group IIb and involving a pathogenic gene, was inherited from a normal parent and was therefore considered to be of unclear significance (Table 2B).

Twenty other CNVs were classified as familial variants (group IIc, Additional file 2: Table S1) that were most likely benign. Eleven were microduplications that ranged between 47 and 1,612 kb. One inherited microdeletion from a healthy mother encompassed intron 4 of the *AUTS2* gene.

Thirty-nine CNVs, above the limited threshold set for the detection of true positive CNVs but with no parental DNA available for further investigation, were also classified as variants of uncertain clinical significance (VOUS) (group IId, Additional file 3: Table S2), of which 84.6% (33/39) were microduplications.

The arrays were also analyzed for significant regions of absence of heterozygosity in search for uniparental disomy, mosaicism, and autosomal recessive pathogenicity in patients with consanguineous parents. Our results did not suggest uniparental disomy or mosaicism but confirmed the high rate of consanguineous marriage in Lebanon: 42 of the 149 patients (28.2%) were confirmed as being born to closely related parents and had greater than 66 Mb of ROH, which correlates with an estimate of F = 1/32. Three of these patients had an ROH size equivalent to that of the theoretical coefficient of inbreeding, F = 1/4, ten patients had an estimated F = 1/8, 22 probands had F = 1/16, and 7 patients had F = 1/32 (Additional file 4: Table S3).

## Discussion

Herein, we present our results from the first Lebanese CMA study investigating the involvement of CNVs in patients with ID/DD. We validated the Cyto2.7 M array platform with a confirmation of 99 identified CNVs using Quantitative PCR (Q-PCR). This resulted in the determination of a clinical threshold of 62 kb with at least 49 consecutive markers. Most abnormalities that do not meet these requirements could not be confirmed and their presence is due to the “cleanness” of the signal detected from the array. This confirms results from previous studies mentioning the importance of taking into consideration array Quality Control parameters [13]. However, the genomic content should be checked in all CNVs as it points to candidate genes that could be missed when applying any threshold. An improvement of the reliability of small CNVs in newer arrays is then required and was applied in the CytoScan HD array by enriching the number and type of markers in a region (oligonucleotide and SNP probes) [14].

Our results indicate an overall diagnostic yield of 12.1%, a value in agreement with previous studies obtaining a detection rate between 10% and 20% [10,15]. Different array types from the same company, like Affymetrix 500 K SNP array are used with a close threshold of 100 kb and also obtain similar diagnostic yield [16]. We confirmed, once more, the pathogenic effect of CNVs belonging to group I. However, we found new genotype/phenotype correlations in three patients, with 1q44, 10q26.11-q26.13, or 16q22.3 interstitial microdeletions, belonging to group I. We reviewed reports of patients with aberrations that overlap these CNVs and propose a variety of new findings for these 3 aberrations.

### 1q44 microdeletion

Patient P2, a 48-month-old female with ID, DD, and facial dysmorphisms (Table 1), was found to have two *de novo* microdeletions involving the 1q44ter region and exon 5 of *JARID2*. The deletion of 1q44 is a recognizable clinical disorder characterized by short stature, DD, ID, microcephaly, facial dysmorphism, variable types of seizure, and partial to complete agenesis of the corpus callosum (ACC) [17-19]. Moreover, deletions of *JARID2* are associated with cognitive impairment and facial features such as prominent supraorbital ridges, deep set eyes, dark infraorbital circles, and midface hypoplasia [20,21]. When carefully examined, patient P2 has the same facial features noted in patients with both deletions, *JARID2* deletion and the 1q44 syndrome.

Although the 1q44 syndrome forms a recognizable phenotype, the disentangling of the genetic causes of seizure, ACC, and microcephaly has been challenging. To explain these features, several critical regions in the 1q44 deletions were defined; the *AKT3* gene has been proposed to be responsible for microcephaly, and the *ZNF238* gene (also named *ZBTB18*) to have a potential role in ACC [22,23]. In patient P2, neither of these genes was deleted; however, she had microcephaly. Ballif et al. proposed a critical region containing three genes: *COX20* with no known relevant function, *HRNUPU* and *HRNUPU-AS1.* The last two when mutated*,* are thought to be responsible for seizures with the occurrence of the first seizure not exceeding 4 years of age [22,24]. The deletion of *HRNUPU*, which is involved in embryonic brain development, is most likely pathogenic because of its haploinsufficiency. Its antisense transcript, HRNUPU-AS1, has been found to affect the expression of HRNUPU [25]. Our patient’s deletion (Additional file 1: Figure S1) overlapped the critical region defined by Ballif et al. encompassing the seizure-causing genes proposed in the literature, although the patient had microcephaly but no seizures. Seizures may not have yet started in the proband studied here, although seizure onset occurs early in the described patients, making future seizures very unlikely. The deletion may also have incomplete penetrance or variable expressivity. Finally, a combination of the 1q44 deletion and the 20 kb deletion of *JARID2*’s exon 5 might explain the patient’s phenotype and an epistatic relationship may exist between the copy number imbalances, exacerbating the proband’s intellectual impairment and leading to the modification of the patient’s clinical features.

### 10q26.11-q26.13 microdeletion

Patient P8 was found to have an interstitial 4,570 kb *de novo* 10q26.11-q26.13 microdeletion. This deleted region and those of some previously described patients with an interstitial 10q26 do not include the minimal critical region (MCR) previously described by Yatsenko et al. [26-32]; however they share similar clinical findings: ID, growth and psychomotor retardation, and microcephaly. We narrowed down the smallest region of overlap responsible for these common features to chr10:122,878,779-124,072,142. This region contains 5 genes: *NSMCE4A, ATE1, TACC2, BTBD16,* and *FGFR2*.

Genital anomalies have been also associated with the deletion of the 10q25.3-q26.1 segment [26]. We suggest the common region 10q26.12 to be responsible for these defects and reduce the previously critical region to a region including only *WDR1* and *PPAPDC1A* (article accepted in AMJG).

### 16q22.3 microdeletion

The 16q22.3 microdeletion was found in patient P15, who was known to have an apparently balanced translocation on standard karyotype between chromosome 1 and 16. Published reports of chromosome 16 microdeletions are very rare. Few cases involve the 16q22.3 region deleted in patient P15 [33-40]. These patients show similar characteristics involving cleft palate, ID, and psychomotor retardation. The deletion described here reduces the smallest region of overlap to 2,262 kb and suggests that the absence of a kidney and the presence of clubbed feet in the patient described by Natt and colleagues [34] as well as congenital heart defects are not caused by the absence of genes present in the overlapping region.

One microimbalance belonging to group IIb was inherited from the healthy parent of patient P24. It is a 64 kb heterozygous deletion affecting exons 9 to 13 of *ATRNL1*. A 325 kb deletion adjacent to this gene, described by Stark et al., implicates this gene in cognitive impairment, autism and several dysmorphic features. *ATRNL1* is involved in the regulation of energy homeostasis by binding to melanocortins [41,42]. The two patients have some common characteristics (Table 3), especially autistic traits, skeletal abnormalities, and ID. However, the presence of the deletion in the healthy mother of patient 24 makes it difficult to assess the clinical significance of this CNV. Therefore, sequencing of *ATRNL1* was performed but no point mutation was found. This CNV can then be considered pathogenic with an incomplete penetrance or simply classified as benign. A study with a larger number of controls is therefore required because only intronic deletions were found in our control database (10 CNVs, all in intron 26).

**Table 3:** **A comparison between two patients with a heterozygous deletion of** ***ATRNL1***

	**Present case P24**	**Stark et al.**
Cardiac problems at birth	-	Small muscular ventricular septal defect at birth but closed spontaneously
Poor suck & hypotonia first weeks of life	-	+
Prominent forehead	Small	+
Epicanthal folds	-	+
Arched eyebrows	+	+
Long eyelashes	+	
Eye problems	-	+
Small mouth	-	+
Small and squared ears	-	+
Upslanting palpebral fissures	+	
Flat pillar of the nose, wide nose	+	-
Fingers/toes abnormalities	Short second toe	2/3 toe syndactyly
Skeletal abnormalities	Syringomyelia	Radioulnar synostosis
Delay	Severe	Moderate, no developmental regression
Walked at the age of	30 months	24 months
Ataxic gait	+	+
Others		eczema

Five CNVs were found in 42 probands with consanguineous parents. This confirms previous studies that showed the importance of microarrays in the identification of causes of ID/DD in probands with consanguineous parents [43]. These CNVs have a pick-up rate of 3.4% (5/149). Three of them were new homozygous deletions: a deletion of the *PTPRD* gene in patient P20 and the two other CNVs characterizing two new phenotypes in patients P22 and P23. Although the clinical significance of the three CNVs is still unclear, they were considered potentially causative because they followed a pattern of autosomal recessive inheritance. A search for other patients with similar phenotypes is necessary to accurately classify these CNVs.

Additionally, we looked for the presence of parental consanguinity suggestive of autosomal recessive disorders. We therefore compared the estimated coefficient of inbreeding to the coefficient deduced from the pedigree of each family, and interestingly, we noticed the occurrence of significant deviations from theoritical values. In 17/42 patient (40.4%), a higher degree of relationship than shown by their pedigree was observed. This variation is due to multiple loops of consanguinity or multiple generations of inbreeding observed within the Lebanese community [44], which complicates the estimation of the degree of relationship. These cases were marked for further investigation with the aim of sequencing candidate genes within the ROH regions.

Finally, we established an internal database for polymorphic CNVs to help further studies discriminate between rare polymorphisms and disease-associated variants (data not shown).

## Conclusions

In conclusion, this is the first Lebanese study on ID/DD patients. It has provided supporting evidence that whole-genome analysis is a powerful method for uncovering chromosomal imbalances and genomic rearrangements, regardless of consanguinity in the parents of patients and despite the challenge presented by analyzing some CNVs.

## Methods

### Ethical statement

This study was carried out with protocols approved by the Institutional Review Board (IRB) on human experimentation at Saint Joseph University.

Patients and controls from all regions of Lebanon were recruited through the Medical Genetics Unit of Saint Joseph University over a period of three years. Approval for the study and informed written consent were obtained from legally authorized patient representatives and the 99 healthy subjects.

### Cohort

A total of 149 Lebanese children (88 boys and 61 girls) with moderate to severe ID associated with developmental delay and/or congenital abnormalities (CA) of unknown origin were analyzed using the Affymetrix Cyto 2.7 M platform. Known syndromes were eliminated using karyotyping, subtelomere FISH, MLPA, and/or fragile X testing. Copy number analysis was performed for 99 healthy Lebanese individuals using two types of arrays, SNP 6.0 and Cyto 2.7.

### Chromosomal microarray analysis-based technologies

#### Cyto 2.7M

Genomic DNA, isolated from peripheral blood samples using the salting-out technique, was amplified, purified, fragmented, denatured, and then hybridized into the Cyto 2.7 M, following the Affymetrix® standard protocol.

A single array has a high density of 2,361,876 non-polymorphic markers and 400,103 SNP markers, with whole-genome backbone coverage of ~1 kb spacing.

The analysis of scanned chips was performed using the Affymetrix Chromosome Analysis Suite software (ChAS v.1.0.1). The software initiates studies on arrays of which the Median Absolute Pairwise Difference score (MAPD), the waviness segment count, and the SNPQC meet the Quality Control (QC) criteria set by the manufacturer: MAPD < 0.27; SNPQC > 1.1; Wav Seg Count ≤ 30 [45]. The annotation file used in our analysis can be found on the Affymetrix website, listed as ArrayNA30.1 (hg18).

#### SNP 6.0

This array consists of 906,600 SNP probes and 900,000 non-polymorphic oligonucleotides used for detecting CNVs with an average spacing of 0.7 kb. The preparation and hybridization of DNA samples were performed following the Affymetrix® 6.0 standard protocol.

### Assessment of array parameters

Assessing the existence and causality of the small CNVs identified by this high-resolution platform was very challenging and required the consideration of array parameters. We selected ninety-nine random CNVs, with no threshold, for validation by quantitative PCR (Q-PCR). Our results (data not shown but available upon request) prompted us to select a threshold of 62 kb with at least 49 consecutive markers, which we further utilized to filter the CNV analysis results.

### Detection of parental disomy and consanguinity

Large ROH observed on a single chromosome can be suggestive of parental disomy (≥10 Mb); however, when distributed throughout the genome, large ROHs are indicative of a consanguineous relationship between the patient’s parents. Small stretches of homozygosity < 3 Mb were ignored because they are common even in outbred populations [46].

To detect possible parental consanguinity, we compared the patient’s ROH size, calculated on several chromosomes (sum of ROH ≥ 3 Mb), with the theoretical ROH size, estimated by multiplying the 2,867,732,772-base total size of the autosomal haploid genome (NCBI Build 36.1 assembly (2006)) by the theoretical value of the coefficient of inbreeding [47]. We also defined a variable range of expected ROH size by using the mid-line between theoretical average sizes as used by Fan et al. (Additional file 5: Table S4) [43].

### Quantitative PCR (Q-PCR)

The array findings were confirmed and their *de novo* and inherited status were distinguished using Q-PCR with an ABI Prism 7500 system (Applied Biosystems, Foster City, CA, USA) using fluorescent SYBR Green dye (ABI). Specific primers targeting genes or intronic sequences were designed using Primer Express 3 Software (ABI).

PCR was performed in a 20 μl reaction volume containing 10 μl Power SYBR-Green PCR Master Mix (ABI), 10 pmol forward and reverse primers, and 10 ng of genomic DNA. The reaction cycling conditions were 95°C for 10 min, followed by 40 cycles of 95°C for 15 sec and 60°C for 1 minute. Each sample was run in triplicate for the quantification of the expression level of a target gene and compared to the expression level of two endogenous genes.

Data evaluation was carried out using the ABI Prism Sequence Detection System (SDS) using the comparative ΔΔ threshold cycle number (Ct) method. To exclude the presence of non-specific products, a melting-curve analysis of the products was performed after completion of the amplification.

### Workflow for selecting CNVs

To assess the clinical significance of the detected CNVs, we followed the recommended steps from Miller et al. and Buysse et al. [10,47,48].

All imbalances found at least twice in the Database of Genomic Variants (DGV) and our internal database of healthy individuals were considered to be benign and excluded from further analysis. CNVs under the selected threshold, as well as those that did not involve genes or miRNAs, were also excluded. The remaining CNVs were classified into groups.

Group I contains pathogenic CNVs overlapping critical regions of known microdeletions or microduplications and/or involving genes already described as causing a phenotype, especially ID. These CNVs are found in the publicly available DECIPHER (http://decipher.sanger.ac.uk) and ISCA (www.clinicalgenome.org) databases and in published literature such as the Catalogue of Unbalanced Chromosome Aberrations in Man [9].

Group II contains genomic imbalances classified as being variants of uncertain clinical significance because of their unclear possible pathogenicity. Parental studies were mandatory for the classification of these CNVs. VOUS were grouped into four categories: Group IIa contains rare, likely pathogenic CNVs that mostly occur *de novo* and includes genes with a possible correlation to the phenotype (abnormal with a low recurrence risk); Group IIb corresponds to CNVs for which clinical interpretation remains uncertain, even after parental studies, owing to variable expressivity or incomplete penetrance; Group IIc includes all inherited CNVs, also called familial variants, that were considered to be benign; and Group IId contains VOUS that could not be further tested owing to the absence of parental DNA.

### DNA sequencing

The coding sequences of *ATRNL1* were sequenced after DNA amplification by PCR (NM-207303). Primers were designed using Primer 3 (http://frodo.wi.mit.edu) and OLIGOS v.9.3, and checked for specificity using BLAST (http://www.ncbi.nlm.nih.gov/blast/bl2seq/wblast2.cgi). PCR reactions were performed using Taq DNA polymerase (Invitrogen Life Technologies, Carlsbad, Calif., USA). PCR products from genomic DNA were purified using the illustra TM GFX PCR DNA and Gel Band Purification Kit (GE Healthcare, Buckinghamshire, UK), and sequenced using the BigDye _ Terminator v1.1 Cycle Sequencing Kit (Applied Biosystems, Foster City, Calif., USA) under standard conditions. The labeled products were subjected to electrophoresis on an Applied Biosystems Genetic Analyzer sequencing system.

## Additional files

Additional file 1: Figure S1. Microdeletion 1q44 and haploinsufficiency of *HNRNPU* gene [16-18,44-46]. Additional file 2: Table S1. Group IIc, CNVs likely benign. Additional file 3: Table S2. CNV of uncertain clinical significance with a threshold at 62 kb and 49 Markers. Additional file 4: Table S3. Calculation of the expected ROH size range using the 2,867,732,772 bases total size of the autosomal haploid genome (NCBI Build 36.1 assembly (2006)) multiplied by the theoretical value of the coefficient of inbreeding. Additional file 5: Table S4. Calculation of the expected ROH size using the 2,867,732,772 bases total size of the autosomal haploid genome (NCBI Build 36.1 assembly (2006)) multiplied by the theoretical value of the coefficient of inbreeding.

## References

[1] Schalock RL, Borthwick-Duffy SA, Bradley VJ, Buntinx WHE, Coulter DL, Craig EM, et al. Intellectual disability: definition, classification, and systems of supports. 11th ed. American Association on Intellectual and Developmental Disabilities; 2010.

[2] OMS | Chapitre 2: Impact des troubles mentaux et du comportement http://www.who.int/whr/2001/chapter2/fr/index4.html

[3] Roeleveld N, Zielhuis GA, Gabreëls F (1997). The prevalence of mental retardation: a critical review of recent literature. Dev Med Child Neurol.

[4] Rauch A, Hoyer J, Guth S, Zweier C, Kraus C, Becker C (2006). Diagnostic yield of various genetic approaches in patients with unexplained developmental delay or mental retardation. Am J Med Genet A.

[5] Scior K (2011). Public awareness, attitudes and beliefs regarding intellectual disability: a systematic review. Res Dev Disabil.

[6] Azar M, Badr LK (2010). Predictors of coping in parents of children with an intellectual disability: comparison between Lebanese mothers and fathers. J Pediatr Nurs.

[7] Sagoo GS, Butterworth AS, Sanderson S, Shaw-Smith C, Higgins JPT, Burton H (2009). Array CGH in patients with learning disability (mental retardation) and congenital anomalies: updated systematic review and meta-analysis of 19 studies and 13,926 subjects. Genet Med Off J Am Coll Med Genet.

[8] Inlow JK, Restifo LL (2004). Molecular and comparative genetics of mental retardation. Genetics.

[9] Verloes A, Héron D, Billette de Villemeur T, Afenjar A, Baumann C, Bahi-Buisson N (2012). Stratégie d’exploration d’une déficience intellectuelle inexpliquée. Arch Pédiatrie.

[10] Miller DT, Adam MP, Aradhya S, Biesecker LG, Brothman AR, Carter NP (2010). Consensus statement: chromosomal microarray is a first-tier clinical diagnostic test for individuals with developmental disabilities or congenital anomalies. Am J Hum Genet.

[11] Rodríguez-Revenga L, Vallespín E, Madrigal I, Palomares M, Mur A, García-Miñaur S (2013). A parallel study of different array-CGH platforms in a set of Spanish patients with developmental delay and intellectual disability. Gene.

[12] Chouery E, Choucair N, Abou Ghoch J, El Sabbagh S, Corbani S, Mégarbané A (2013). Report on a patient with a 12q24.31 microdeletion inherited from an insulin-dependent diabetes mellitus father. Mol Syndromol.

[13] Qiao Y, Tyson C, Hrynchak M, Lopez-Rangel E, Hildebrand J, Martell S (2013). Clinical application of 2.7 M Cytogenetics array for CNV detection in subjects with idiopathic autism and/or intellectual disability. Clin Genet.

[14] Uddin M, Thiruvahindrapuram B, Walker S, Wang Z, Hu P, Lamoureux S, et al. A high-resolution copy-number variation resource for clinical and population genetics. Genet Med Off J Am Coll Med Genet. 2014; doi:10.1038/gim.2014.178 10.1038/gim.2014.178 PMC4752593 25503493

[15] Shoukier M, Klein N, Auber B, Wickert J, Schröder J, Zoll B (2013). Array CGH in patients with developmental delay or intellectual disability: are there phenotypic clues to pathogenic copy number variants?. Clin Genet.

[16] McMullan DJ, Bonin M, Hehir-Kwa JY, de Vries BBA, Dufke A, Rattenberry E (2009). Molecular karyotyping of patients with unexplained mental retardation by SNP arrays: a multicenter study. Hum Mutat.

[17] Merritt JL, Zou Y, Jalal SM, Michels VV (2007). Delineation of the cryptic 1qter deletion phenotype. Am J Med Genet A.

[18] Mankinen CB, Sears JW, Alvarez VR (1976). Terminal (1)(q43) long-arm deletion of chromosome no. 1 in a three-year-old female. Birth Defects Orig Artic Ser.

[19] Caliebe A, Kroes HY, van der Smagt JJ, Martin-Subero JI, Tönnies H, van ’t Slot R (2010). Four patients with speech delay, seizures and variable corpus callosum thickness sharing a 0.440 Mb deletion in region 1q44 containing the HNRPU gene. Eur J Med Genet.

[20] Carayol J, Schellenberg GD, Dombroski B, Genin E, Rousseau F, Dawson G (2011). Autism risk assessment in siblings of affected children using sex-specific genetic scores. Mol Autism.

[21] Barøy T, Misceo D, Strømme P, Stray-Pedersen A, Holmgren A, Rødningen OK (2013). Haploinsufficiency of two histone modifier genes on 6p22.3, ATXN1 and JARID2, is associated with intellectual disability. Orphanet J Rare Dis.

[22] Ballif BC, Rosenfeld JA, Traylor R, Theisen A, Bader PI, Ladda RL (2012). High-resolution array CGH defines critical regions and candidate genes for microcephaly, abnormalities of the corpus callosum, and seizure phenotypes in patients with microdeletions of 1q43q44. Hum Genet.

[23] Shimojima K, Okamoto N, Suzuki Y, Saito M, Mori M, Yamagata T (2012). Subtelomeric deletions of 1q43q44 and severe brain impairment associated with delayed myelination. J Hum Genet.

[24] Van Bon BWM, Koolen DA, Borgatti R, Magee A, Garcia-Minaur S, Rooms L (2008). Clinical and molecular characteristics of 1qter microdeletion syndrome: delineating a critical region for corpus callosum agenesis/hypogenesis. J Med Genet.

[25] Poot M, Kas MJ (2013). Antisense may make sense of 1q44 deletions, seizures, and HNRNPU. Am J Med Genet A.

[26] Yatsenko SA, Kruer MC, Bader PI, Corzo D, Schuette J, Keegan CE (2009). Identification of critical regions for clinical features of distal 10q deletion syndrome. Clin Genet.

[27] Mardo V, Squibb EE, Braverman N, Hoover-Fong JE, Migeon C, Batista DAS (2008). Molecular cytogenetic analysis of a de novo interstitial deletion of chromosome 10q (q25.3q26.13) in a male child with ambiguous genitalia: Evidence for a new critical region for genital development. Am J Med Genet A.

[28] Miller ND, Nance MA, Wohler ES, Hoover-Fong JE, Lisi E, Thomas GH (2009). Molecular (SNP) analyses of overlapping hemizygous deletions of 10q25.3 to 10qter in four patients: evidence for HMX2 and HMX3 as candidate genes in hearing and vestibular function. Am J Med Genet A.

[29] Chitkara R, Rajani A, Bernstein J, Shah S, Hahn J, Barnes P (2011). Newborn with Prenatally Diagnosed Choroidal Fissure Cyst and Panhypopituitarism and Review of the Literature. Am J Perinatol Rep.

[30] Irving M, Hanson H, Turnpenny P, Brewer C, Ogilvie CM, Davies A (2003). Deletion of the distal long arm of chromosome 10; is there a characteristic phenotype? A report of 15 de novo and familial cases. Am J Med Genet A.

[31] Chang Y-T, Chou I-C, Wang C-H, Chin Z-N, Kuo H-T, Lin C-C (2013). Chromosome 10q deletion del (10)(q26.1q26.3) is associated with cataract. Pediatr Neonatol.

[32] Rooney DE, Williams K, Coleman DV, Habel A (1989). A case of interstitial deletion of 10q25.2–q26.1. J Med Genet.

[33] Fujiwara M, Yoshimoto T, Morita Y, Kamada M (1992). Interstitial deletion of chromosome 16q: 16q22 is critical for 16q- syndrome. Am J Med Genet.

[34] Natt E, Westphal EM, Toth-Fejel SE, Magenis RE, Buist NR, Rettenmeier R (1987). Inherited and de novo deletion of the tyrosine aminotransferase gene locus at 16q22.1–q22.3 in a patient with tyrosinemia type II. Hum Genet.

[35] Cooke A, Tolmie J, Darlington W, Boyd E, Thomson R, Ferguson-Smith MA (1987). Confirmation of a suspected 16q deletion in a dysmorphic child by flow karyotype analysis. J Med Genet.

[36] Natt E, Magenis RE, Zimmer J, Mansouri A, Scherer G (1989). Regional assignment of the human loci for uvomorulin (UVO) and chymotrypsinogen B (CTRB) with the help of two overlapping deletions on the long arm of chromosome 16. Cytogenet Cell Genet.

[37] Edelhoff S, Maier B, Trautmann U, Pfeiffer RA (1991). Interstitial deletion of 16(q13q22) in a newborn resulting from a paternal insertional translocation. Ann Génétique.

[38] Callen DF, Eyre H, Lane S, Shen Y, Hansmann I, Spinner N (1993). High resolution mapping of interstitial long arm deletions of chromosome 16: relationship to phenotype. J Med Genet.

[39] Khan A, Hyde RK, Dutra A, Mohide P, Liu P (2006). Core binding factor beta (CBFB) haploinsufficiency due to an interstitial deletion at 16q21q22 resulting in delayed cranial ossification, cleft palate, congenital heart anomalies, and feeding difficulties but favorable outcome. Am J Med Genet A.

[40] Yamamoto T, Dowa Y, Ueda H, Kawataki M, Asou T, Sasaki Y (2008). Tetralogy of Fallot associated with pulmonary atresia and major aortopulmonary collateral arteries in a patient with interstitial deletion of 16q21-q22.1. Am J Med Genet A.

[41] Stark Z, Bruno DL, Mountford H, Lockhart PJ, Amor DJ (2010). De novo 325 kb microdeletion in chromosome band 10q25.3 including ATRNL1 in a boy with cognitive impairment, autism and dysmorphic features. Eur J Med Genet.

[42] Haqq AM, René P, Kishi T, Khong K, Lee CE, Liu H (2003). Characterization of a novel binding partner of the melanocortin-4 receptor: attractin-like protein. Biochem J.

[43] Fan Y-S, Ouyang X, Peng J, Sacharow S, Tekin M, Barbouth D (2013). Frequent detection of parental consanguinity in children with developmental disorders by a combined CGH and SNP microarray. Mol Cytogenet.

[44] Jalkh N, Sahbatou M, Chouery E, Megarbane A, Leutennegger A-L, Serre J-L. Genome-wide inbreeding estimation within Lebanese communities using SNP arrays. Eur J Hum Genet EJHG. 2014; doi:10.1038/ejhg.2014.246 10.1038/ejhg.2014.246 PMC4592074 25424710

[45] Comparison for cytogenetics array platforms hardware and software for use in identifying copy number aberrations in constitutional disorders http://www.ngrl.org.uk/Wessex/downloads/pdf/NGRLW_aCGH_1%5B1%5D.0.pdf

[46] Rehder C, David K, Hirsch B, Toriello H, Wilson C, Kearney H (2013). American College of Medical Genetics and Genomics: standards and guidelines for documenting suspected consanguinity as an incidental finding of genomic testing. Genetics in medicine.

[47] Straub RE, MacLean CJ, Ma Y, Webb BT, Myakishev MV, Harris-Kerr C (2002). Genome-wide scans of three independent sets of 90 Irish multiplex schizophrenia families and follow-up of selected regions in all families provides evidence for multiple susceptibility genes. Mol Psychiatry.

[48] Buysse K, Delle Chiaie B, Van Coster R, Loeys B, De Paepe A, Mortier G (2009). Challenges for CNV interpretation in clinical molecular karyotyping: lessons learned from a 1001 sample experience. Eur J Med Genet.

