# Supplementary material for: Contribution of copy number variants (CNVs) to congenital, unexplained intellectual and developmental disabilities in Lebanese patients
Source: Mol Cytogenet. 2015 Apr 9;8:26. doi: 10.1186/s13039-015-0130-y (PMC4411788; doi:10.1186/s13039-015-0130-y)
Supplement: Additional file 1: Figure S1. — Microdeletion 1q44 and haploinsufficiency of HNRNPU gene [16-18,44-46]. [file 13039_2015_130_MOESM1_ESM.docx]

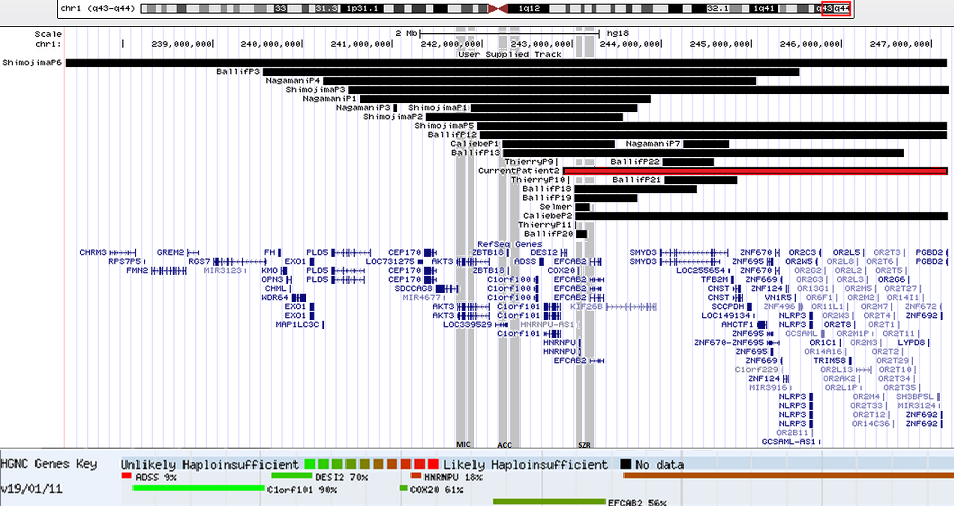


**Fig. S1.** Microdeletion 1q44 and haploinsufficiency of *HNRNPU* gene.

The upper figure shows the deletion region of patient 2, and of the patients described by Caliebe et al.[16], Ballif et al. [17], Shimojima et al.[18], Nagamani et al.[44], Thierry et al. [45], Selmer et al. [46] depicted on a genome map from UCSC. The region responsible for microcephaly (MIC), ACC, and SZR as predicted by Ballif et al. [17] are shown by grey rectangles.

The lower figure shows the haploinsufficiency of *HNRNPU* gene.
