# Supplementary material for: Contribution of copy number variants (CNVs) to congenital, unexplained intellectual and developmental disabilities in Lebanese patients
Source: Mol Cytogenet. 2015 Apr 9;8:26. doi: 10.1186/s13039-015-0130-y (PMC4411788; doi:10.1186/s13039-015-0130-y)
Supplement: Additional file 2: Table S1. — Group IIc, CNVs likely benign. [file 13039_2015_130_MOESM2_ESM.docx]

**Table S1:** Group IIc, CNVs likely benign.

ND: inheritance not determined.

* Same or overlapping CNV found as inherited in another patient of a different cohort.

| **Chromosome** | **Minimal breakpoint** | **Type of CNV** | **Gene tested**  **by Q-PCR** | **Size (kb)** | **Inheritance** |
| --- | --- | --- | --- | --- | --- |
| 1p33 | 49,294,319-49,511,488 | Loss | *AGBL4* | 217 | Maternal |
| 2q24.1 | 157,726,958-158,126,211 | Gain | *GALNT5* | 399 | Maternal |
| 2q12.3 | 106,791,311-107,371,178 | Gain | *ST6GAL2* | 505 | Maternal |
| 2p15 | 61,174,369-61,332,922 | Gain | *USP34* | 158 | Maternal |
| 4q31.3 | 151,203,490-151,211,576 | Loss | 5'UTR 8kb of *DCLK2* | 8 | Paternal |
| 4q13..3 | 71,195,457-71,318,007 | Loss | *C4orf35* | 122 | ND* |
| 7q11.22 | 69,330,517-69,406,419 | Loss | *AUTS2* | 112 | Maternal |
| 7q31.31 | 120,437,909-120,706,017 | Gain | *C7orf58* | 268 | Maternal |
| 8p21.3 | 19,424,017-19,665,441 | Gain | *SH2D4A* | 409 | Paternal |
| 10q11.22 | 49,330,726-49,813,818 | Gain | *ARHGAP22* | 499 | Paternal |
| 12p12.3 | 15,983,482-16,186,392 | Loss | *DERA* | 202 | Maternal |
| 12q24.31 | 119,633,574-120,613,673 | Loss | *HNF1A* | 980 | Paternal |
| 14q21.1 | 38,757,604-38,809,076 | Gain | *MIA2* | 51 | Paternal |
| 17p12 | 15,498,255-15,765,384 | Gain | *TBC1D26* | 267 | Maternal |
| 19q13.21 | 40,264,011-40,349,468 | Gain | *LGI4* | 85 | Paternal |
| 19q13.31 | 49,480,029-49,513,301 | Loss | *ZNF235* | 33 | Maternal |
| 19q12 | 32,972,439-33,154,112 | Loss | *Loc148189* | 182 | Paternal |
| Xq13.3 | 74,685,923-74,748,742 | Loss | 5'UTR 26 kb of *ZDHHC15* | 62 | Maternal |
| Xq28 | 148,493,888-148,541,566 | Gain | *TMEM185A* | 47 | ND* |
| Xp22.31 | 6,478,413-8,090,906 | Gain | *HDHD1A* | 1,612 | Paternal |
