# Supplementary material for: Contribution of copy number variants (CNVs) to congenital, unexplained intellectual and developmental disabilities in Lebanese patients
Source: Mol Cytogenet. 2015 Apr 9;8:26. doi: 10.1186/s13039-015-0130-y (PMC4411788; doi:10.1186/s13039-015-0130-y)
Supplement: Additional file 3: Table S2. — CNV of uncertain clinical significance with a threshold at 62 kb and 49 Markers. [file 13039_2015_130_MOESM3_ESM.docx]

| **Patient's Gender**  **Table S2:** CNV of uncertain clinical significance with a threshold at 62 kb and 49 Markers. | **CN State** | **Type** | **Chromosome** | **Minal breakpoints (bp)** | **Cytoband Start** | **Marker Count** | **Size (kbp)** | **Genes** |
| --- | --- | --- | --- | --- | --- | --- | --- | --- |
| F | 3 | Gain | 1 | 235,070,861- 235,589,821 | q43 | 630 | 518 | *MTR, RYR2* |
| F | 3 | Gain | 2 | 70,913,413- 70,998,576 | p13.3 | 56 | 85 | *VAX2, CD207* |
| F | 3 | Gain | 2 | 95,299,684- 95,387,392 | q11.1 | 70 | 87 | *PROM2, KCNIP3* |
| F | 3 | Gain | 2 | 31,114,829- 31,217,877 | p23.1 | 111 | 103 | *GALNT14* |
| M | 4 | Gain | 2 | 135,414,122- 136,095,860 | q21.3 | 577 | 681 | *RAB3GAP1, YSK4, ZRANB3, R3HDM1, CCNT2* |
| M | 3 | Gain | 4 | 39,757,547- 39,920,522 | p14 | 146 | 162 | *N4BP2, RHOH* |
| M | 3 | Gain | 4 | 106,524,103- 107,007,500 | q24 | 483 | 483 | *EEF1AL7, FLJ20184, GSTCD, INTS12, PPA2,* |
| M | 3 | Gain | 6 | 109,464,702- 109,534,332 | q21 | 74 | 69 | *C6orf182, SESN1* |
| F | 1 | Loss | 6 | 55,957,183- 56,047,500 | p12.1 | 84 | 90 | *COL21A1* |
| F | 3 | Gain | 6 | 118,839,921- 118,969,839 | q22.31 | 117 | 129 | *C6orf204* |
| M | 1 | Loss | 6 | 56,981,096- 57,136,966 | p12.1 | 164 | 155 | *BEND6, KIAA1586, ZNF451* |
| M | 3 | Gain | 7 | 97,155,069- 97,222,124 | q21.3 | 53 | 67 | *TAC1* |
| M | 3 | Gain | 7 | 34,037,385- 34,113,140 | p14.3 | 103 | 75 | *BMPER* |
| F | 3 | Gain | 7 | 151,233,380- 151,778,702 | q36.1 | 494 | 545 | *CCT8L1, FABP5L3, GALNT11, GALNTL5, MLL3* |
| M | 1 | Loss | 8 | 68,184,113- 68,250,852 | q13.2 | 72 | 66 | *CSPP1* |
| M | 3 | Gain | 8 | 36,820,340- 36,902,122 | p12 | 92 | 81 | *KCNU1, FKSG2* |
| M | 3 | Gain | 8 | 53,760,522- 54,050,456 | q11.23 | 222 | 289 | *RB1CC1* |
| M | 3 | Gain | 8 | 131,980,545- 132,859,278 | q24.22 | 800 | 878 | *ADCY8* |
| F | 3 | Gain | 9 | 119,028,914- 119,743,856 | q33.1 | 766 | 714 | *ASTN2, TLR4* |
| F | 3 | Gain | 10 | 87,839,875- 88,125,252 | q23.1 | 312 | 285 | *GRID1* |
| M | 1 | Loss | 12 | 98,965,472- 99,130,407 | q23.1 | 152 | 164 | *GOLGA2L1, UHRF1BP1L, ACTR6* |
| F | 3 | Gain | 13 | 30,167,022- 30,384,338 | q12.3 | 184 | 217 | *ALOX5AP, C13orf33* |
| M | 3 | Gain | 13 | 35,142,482- 35,242,051 | q13.3 | 62 | 99 | *NBEA, DCLK1* |
| M | 3 | Gain | 13 | 59,861,708- 59,928,594 | q21.2 | 68 | 66 | *TDRD3* |
| F | 3 | Gain | 13 | 110,741,054- 110,805,049 | q34 | 59 | 63 | *C13orf16, ARHGEF7* |
| M | 1 | Loss | 15 | 66,160,315- 66,218,969 | q23 | 70 | 58 | *PIAS1* |
| F | 3 | Gain | 15 | 82,193,703- 82,640,143 | q25.2 | 452 | 446 | *ADAMTSL3, EFTUD1P1, DNM1P41, Loc100505679* |
| F | 3 | Gain | 17 | 9,346,530- 9,403,322 | p13.1 | 74 | 56 | *STX8* |
| M | 3 | Gain | 18 | 27,468,445- 27,535,527 | q12.1 | 63 | 67 | *B4GALT6* |
| F | 3 | Gain | 18 | 71,021,409- 71,102,407 | q22.3 | 73 | 80 | *ZADH2, TSHZ1* |
| F | 3 | Gain | 19 | 63,319,271- 63,400,097 | q13.43 | 73 | 80 | *ZNF274, ZNF329, ZSCAN18* |
| F | 3 | Gain | 20 | 232,074- 403,051 | p13 | 106 | 170 | *NRSN2, RBCK1, SOX12, TBC1D20, TRIB3* |
| M | 3 | Gain | 20 | 25,071,022- 25,189,113 | p11.21 | 90 | 118 | *ENTPD6, LOC284798, PYGB* |
| F | 3 | Gain | 21 | 42,086,174- 42,182,362 | q22.3 | 61 | 96 | *PRDM15* |
| F | 3 | Gain | 21 | 46,570,066- 46,722,310 | q22.3 | 128 | 152 | *DIP2A, PCNT* |
| F | 1 | Loss | 22 | 34,342,753- 34,431,199 | q12.3 | 71 | 88 | *MB, APOL6* |
| F | 2 | Gain | X | 84,013,514- 84,128,910 | q21.1 | 54 | 115 | *UBE2DNL* |
| F | 2 | Gain | X | 6,070,390- 6,139,138 | p22.31 | 67 | 68 | *NLGN4X* |
| F | 2 | Gain | X | 122,663,636- 122,954,011 | q25 | 127 | 290 | *THOC2, XIAP, STAG2* |
