# Supplementary material for: Contribution of copy number variants (CNVs) to congenital, unexplained intellectual and developmental disabilities in Lebanese patients
Source: Mol Cytogenet. 2015 Apr 9;8:26. doi: 10.1186/s13039-015-0130-y (PMC4411788; doi:10.1186/s13039-015-0130-y)
Supplement: Additional file 4: Table S3. — Calculation of the expected ROH size range using the 2,867,732,772 bases total size of the autosomal haploid genome (NCBI Build 36.1 assembly (2006)) multiplied by the theoretical value of the coefficient of inbreeding. [file 13039_2015_130_MOESM4_ESM.docx]

**Table S3:** Calculation of the expected ROH size range using the 2,867,732,772 bases total size of the autosomal haploid genome (NCBI Build 36.1 assembly (2006)) multiplied by the theoretical value of the coefficient of inbreeding.

NA: not available.

| **Patients** | 1 or P20 | 2 | 3 or P23 | 4 | 5 | 6 | 7 or P24 | 8 | 9 | 10 or P22 | 11 | 12 | 13 | 14 | 15 | 16 | 17 | 18 | 19 | 20 | 21 | 22 | 23 | 24 | 25 | 26 | 27 | 28 | 29 | 30 or P6 | 31 | 32 | 33 | 34 | 35 | 36 | 37 | 38 | 39 | 40 | 41 | 42 |
| --- | --- | --- | --- | --- | --- | --- | --- | --- | --- | --- | --- | --- | --- | --- | --- | --- | --- | --- | --- | --- | --- | --- | --- | --- | --- | --- | --- | --- | --- | --- | --- | --- | --- | --- | --- | --- | --- | --- | --- | --- | --- | --- |
| **Information from pedigree on the coefficient of inbreeding** | 1/16 | 1/16 | 1/16 | 1/16 | 1/16 | 1/16 | 1/16 | 1/64 | NA | 1/8 | NA | 1/16 | 1/8 | 1/16 | 1/9 | 1/16 | 1/16 | 1/16 | 1/12 | 1/10 | 1/16 | 1/16 | 1/16 | 1/64 | 1/64 | 1/16 | 1/16 | 1/16 | 1/16 | 1/32 | 1/11 | 1/16 | 1/32 | 1/64 | 1/16 | 1/64 | 1/16 | 1/16 | 1/16 | NA | 1/16 | 1/16 |
| **Estimated coefficient of inbreeding** | 1/16 | 1/16 | 1/16 | 1/8 | 1/16 | 1/8 | 1/16 | 1/16 | 1/16 | 1/4 | 1/16 | 1/16 | 1/8 | 1/8 | 1/8 | 1/8 | 1/8 | 1/8 | 1/16 | 1/32 | 1/16 | 1/16 | 1/32 | 1/32 | 1/16 | 1/16 | 1/4 | 1/16 | 1/16 | 1/32 | 1/16 | 1/8 | 1/16 | 1/32 | 1/16 | 1/16 | 1/32 | 1/4 | 1/16 | 1/32 | 1/8 | 1/16 |
| **Size of ROH (Mb)** | 173 | 211 | 159 | 271 | 144 | 343 | 199 | 223 | 186 | 570 | 260 | 199 | 345 | 277 | 465 | 296 | 391 | 321 | 234 | 109 | 176 | 213 | 913 | 101 | 139 | 231 | 572 | 242 | 224 | 90 | 251 | 371 | 166 | 101 | 211 | 189 | 81 | 712 | 226 | 100 | 288 | 175 |
