# Supplementary material for: Contribution of copy number variants (CNVs) to congenital, unexplained intellectual and developmental disabilities in Lebanese patients
Source: Mol Cytogenet. 2015 Apr 9;8:26. doi: 10.1186/s13039-015-0130-y (PMC4411788; doi:10.1186/s13039-015-0130-y)
Supplement: Additional file 5: Table S4. — Calculation of the expected ROH size using the 2,867,732,772 bases total size of the autosomal haploid genome (NCBI Build 36.1 assembly (2006)) multiplied by the theoretical value of the coefficient of inbreeding. [file 13039_2015_130_MOESM5_ESM.docx]

**Table S4:** Calculation of the expected ROH size using the 2,867,732,772 bases total size of the autosomal haploid genome (NCBI Build 36.1 assembly (2006)) multiplied by the theoretical value of the coefficient of inbreeding.

| **Degree of relationship** | **Coefficient of inbreeding (F)** | **Theoretic proportion of identical by descent (%)** | **Expected ROH size** | **Range of expected ROH size** |
| --- | --- | --- | --- | --- |
| 1st | 1/4 | 25 | 716 | 537-1074 |
| 2^nd^ | 1/8 | 12.5 | 358 | 270-536 |
| 3rd | 1/16 | 6.25 | 179 | 134-269 |
| 4th | 1/32 | 3.125 | 89 | 66-133 |
